# IFT Proteins Accumulate during Cell Division and Localize to the Cleavage Furrow in Chlamydomonas

**DOI:** 10.1371/journal.pone.0030729

**Published:** 2012-02-06

**Authors:** Christopher R. Wood, Zhaohui Wang, Dennis Diener, James Matt Zones, Joel Rosenbaum, James G. Umen

**Affiliations:** 1 Department of Molecular Cellular and Developmental Biology, Yale University, New Haven, Connecticut, United States of America; 2 Division of Biological Sciences, University of California San Diego, La Jolla, California, United States of America; 3 Donald Danforth Plant Science Center, St. Louis, Missouri, United States of America; University of Texas-Houston Medical School, United States of America

## Abstract

Intraflagellar transport (IFT) proteins are well established as conserved mediators of flagellum/cilium assembly and disassembly. However, data has begun to accumulate in support of IFT protein involvement in other processes elsewhere in the cell. Here, we used synchronous cultures of Chlamydomonas to investigate the temporal patterns of accumulation and localization of IFT proteins during the cell cycle. Their mRNAs showed periodic expression that peaked during S and M phase (S/M). Unlike most proteins that are synthesized continuously during G1 phase, IFT27 and IFT46 levels were found to increase only during S/M phase. During cell division, IFT27, IFT46, IFT72, and IFT139 re-localized from the flagella and basal bodies to the cleavage furrow. IFT27 was further shown to be associated with membrane vesicles in this region. This localization pattern suggests a role for IFT in cell division.

## Introduction

Cilia and flagella (used interchangeably) are organelles used for motility, sensory transduction, and signaling in diverse eukaryotes that include animals, plants and unicells [1]. They are built from an extended ring of nine microtubule doublets (the axoneme) that is templated by basal bodies docked at the plasma membrane. Assembly and maintenance of flagella involve continuous transport of cargo proteins up and down their axonemes by anterograde and retrograde microtubule-based motors in a process termed intraflagellar transport (IFT) [2], [3], [4]. IFT cargo proteins are synthesized in the cell body, loaded onto anterograde IFT complexes, and then carried along the axoneme to the distal tips of flagella where they are assembled. Disassembly also occurs at the tips where proteins that have been removed are transported down axonemes by retrograde IFT complexes [5]. Anterograde and retrograde IFT maintain a regulated steady state balance of assembly and disassembly at the tips of flagella [6], [7], [8], [9].

IFT proteins were first identified in the model biflagellate alga Chlamydomonas reinhardtii [10], [11], [12]. The IFT proteins from Chlamydomonas purify as two sub-complexes, IFT A and IFT B that are required for retrograde and anterograde IFT, respectively. The majority of Chlamydomonas IFT proteins are conserved and have animal homologs, many of which are genetically linked to human ciliopathies [13], [14], [15], [16]. Most IFT proteins in Chlamydomonas are encoded by non-essential genes, some of which were identified through forward genetic screens for flagella-less or bald phenotypes [17].

Apart from their defective flagellar assembly phenotypes, IFT mutants grow nearly as well as wild type cells, a finding that was interpreted to mean that they do not have additional aberrant cell cycle phenotypes [18], [19], [20]. This interpretation is complicated by the fact that, in Chlamydomonas, flagella-less mutants have secondary defects in hatching and can remain trapped in their mother cell wall for several generations after division, forming large clumps, thus precluding detailed analyses of their growth and cell cycle kinetics. Interestingly, using RNAi-based methods, the mammalian IFT88 homolog was shown to be a regulator of the cell cycle in non-ciliated, tissue culture cells [21] and to play a role in spindle orientation [22]. A relationship between flagella length and cell size was reported in IFT knockdowns in Trypanosomes suggesting that IFT proteins and or the flagella might control either growth or cell cycle progression in this flagellate [23]. More recently, at least one IFT complex B subunit, IFT27, a Rab-like GTPase, was found to play a role in both flagellar biogenesis and cytokinesis [24]. While no IFT27 mutants have been identified, RNAi-mediated knockdown of IFT27 or expression of a dominant-negative GFP-IFT27 fusion caused flagellar defects and growth arrest with multinucleated cells, an indicator of failed or aberrant cytokinesis. A second intriguing property of Chlamydomonas IFT27 knockdown strains was a reduction of other IFT proteins from both the A and B subcomplexes suggesting that levels of IFT27 might play a role in coordinating the total levels of IFT proteins [24].

The synthesis of IFT proteins and other flagellar proteins has been examined in Chlamydomonas in the context of flagellar regeneration [25], [26], [27]. Upon deflagellation, mRNAs encoding flagellar proteins, including those of IFT, are rapidly accumulated due to transcriptional activation. A less well understood process of flagella resorption and regeneration occurs during each cell division cycle when basal bodies are redeployed as a part of the mitotic and cytokinetic apparatus [28], [29], [30], [31], [32]. During division, flagella must be removed or resorbed, and failure to do so can result in cell division defects [33], [34], [35], [36]. This pattern of cell cycle correlated ciliogenesis in Chlamydomonas is conserved in mammalian cells with primary cilia, but relatively little is known about how cell division and ciliogenesis might be interdependent [37], [38], [39], [40].

IFT proteins in Chlamydomonas have previously been localized to flagella and in the cell body around basal bodies [5], [11], [41], [42], [43]. IFT52 was also reported at or near the spindle poles, perhaps in association with basal bodies [11], [41], but the possible cytoplasmic localizations of IFT proteins have not been examined thoroughly during the cell cycle [21].

Here, we used synchronous Chlamydomonas cultures to enable the quantitative examination of flagella lengths, IFT mRNA levels, IFT protein levels and IFT protein localization during the cell cycle. We found that IFT complex B mRNAs and proteins are expressed once per cell cycle during M phase and then remain at fixed levels per cell during G1. Interestingly, IFT27 as well as other IFT complex B proteins, and an IFT complex A protein, are relocalized to the cleavage furrow during cytokinesis. Our results provide a framework for understanding the relationship between IFT and the cell cycle, and bolster an emerging theme of alternative roles for IFT proteins outside of ciliogenesis [44], [45].

## Results

### Expression of IFT proteins during the cell cycle

**Figure 1:**
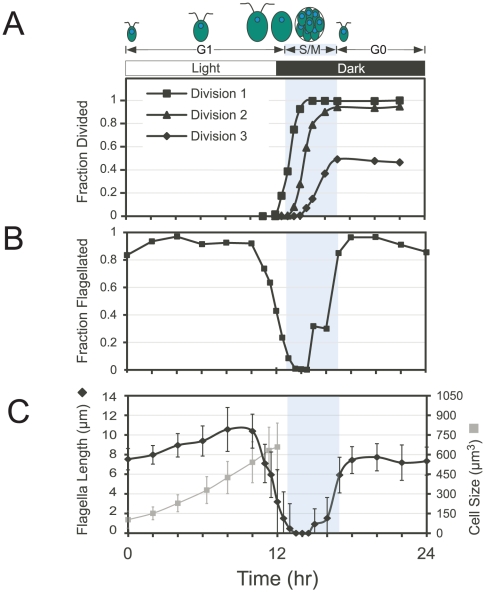
Cell cycle synchrony and flagella dynamics. (A) Top, schematic of cell size and cell cycle progression during G1, S phase and mitosis (S/M), and post-mitosis G0, in phototrophic cultures grown in an alternating light-dark cycle whose phases are shown by the shaded bars. Cells grow during the light phase, resorb flagella, and then divide up to three times in succession to produce daughters that regrow flagella. Bottom, graph of progression through successive cell divisions as assessed by mother cells that completed their first, second and third rounds of division. S/M is marked by light blue shading in this graph and all subsequent graphs. Total cell mass increased by about six-fold during G1 and total cell number increased by about six-fold during S/M. (B) Graph showing fraction of cells with flagella (n = 200). (C) Graph of average flagella length (n = 200) with standard deviation versus average cell size with standard deviation (n≥5000). Cell growth ceases in the dark and was not determined after 12 hours. See also Supporting Text and Fig. S1.

A 12 hr∶12 hr light∶dark cycle was used to induce synchrony in wild type cells that divide by use of a multiple fission cell cycle [46](Fig. 1). In these minimal media cultures (see Materials and Methods) cells grew only during the light phase (G1), enlarging by six to eight-fold (Fig. 1C). Then, at the beginning of the dark phase, cells underwent a division period characterized by two or three rapid rounds of alternating S phase and mitosis (S/M) (Fig. 1A). Just prior to S/M, flagella were resorbed (Fig. 1B, C). Progression through S/M was scored by counting the fraction of cells that were undergoing, or had completed, their first, second or third round of division (Fig. 1A). Cell number increased by about six-fold during S/M. The time period during which division took place is demarcated in all figures with blue shading. Mitotic cells appeared at 13 hours (1 hour into the dark period) and S/M was complete by 17 hours. After completion of S/M, daughter cells entered a G0 (non-growing) state until the next light period. As expected, the flagella rapidly shortened and disappeared just prior to S/M, and then rapidly re-grew after daughter cells completed division (Fig. 1C).

Flagellar lengths were measured continuously and were found to increase gradually as cells progressed through G1 (Fig. 1C). Further examination of different-size cells from asynchronous cultures confirmed a significant positive correlation between cell size and flagellar length (p<0.001) (Text S1 and Figure S1).

### IFT mRNAs during the cell cycle

**Figure 2:**
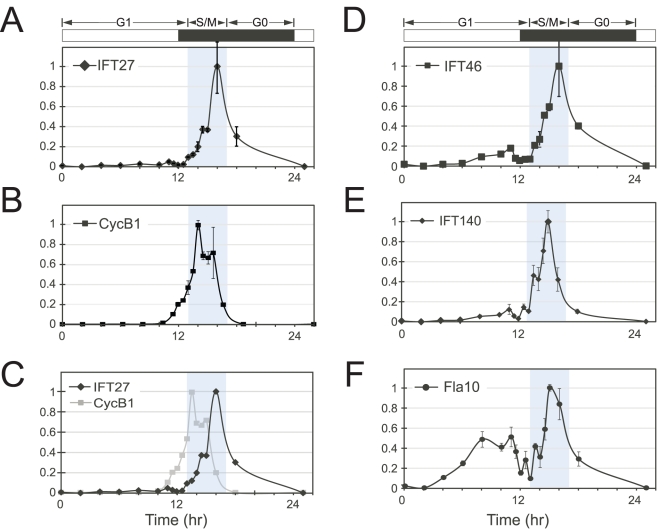
Cell cycle regulation of IFT mRNAs. (A–F) Quantitative RT-PCR from samples taken at different time points during the cell cycle. Samples were normalized first to an internal control (GBLP/CBLP, Genbank ID X53574.1) and then relative to the maximum expression value that was set to 1. The curves for panels B and C are superimposed to show the phase shift in IFT27 expression compared with a message for a representative cell cycle regulatory gene, CYCB1.

We measured the levels of IFT27 mRNA and other mRNAs during the cell cycle by quantitative RT-PCR (qRT-PCR)(Fig. 2, Materials and Methods, Tables S1, S2). IFT27 mRNA levels were found to be strongly regulated, with a burst of expression occurring toward the end of S/M (Fig. 2A). An expression pattern similar to IFT27 was observed for two other IFT mRNAs, IFT46 and IFT140 (Figs. 2D and 2E). This cyclical expression pattern resembled that previously reported for cell cycle mRNAs in Chlamydomonas such as CYCB1 (Fig. 2B) [46], [47]. However, when the plots were superimposed, the peak for IFT mRNA expression was phase shifted by about two hours relative to that of CYCB1 mRNA (Fig. 2C). The mRNA for FLA10, which encodes one subunit of the anterograde kinesin motor for IFT was also found to be cyclically expressed, but exhibited less change than IFT mRNAs between G1 and S/M as indicated by relatively high mRNA levels of FLA10 for most of the G1 time points (Fig. 2F and Table S2). In summary, there are at least two waves of cell cycle mRNA accumulation during S/M, with canonical cell cycle protein mRNAs peaking first, followed by IFT-encoding mRNAs.

### IFT proteins during the cell cycle

**Figure 3:**
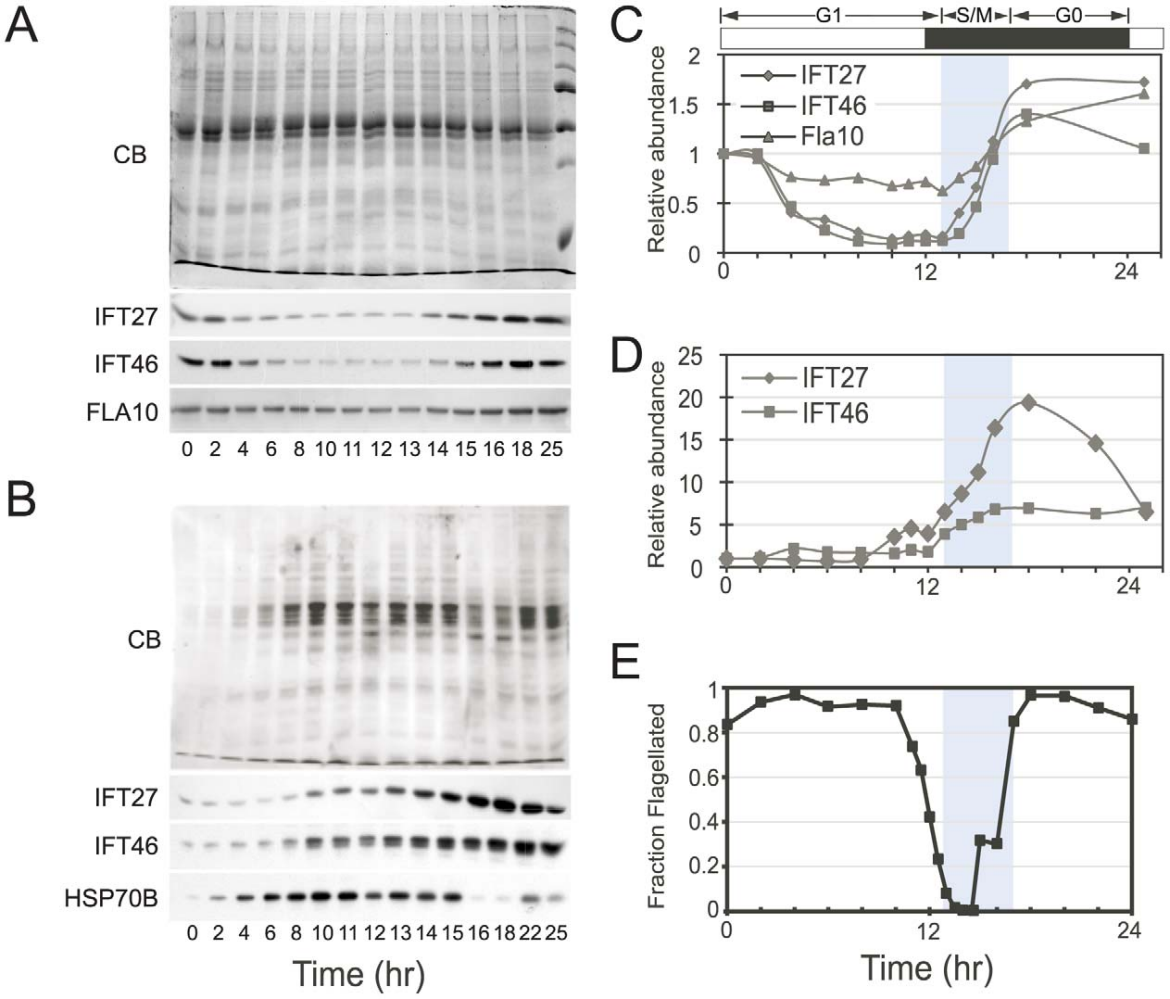
Cell cycle regulation of IFT proteins. (A) Top panel is Coomassie Blue (CB) stained gel of total protein from indicated times. Equal protein was loaded in each lane. The last lane contains molecular weight markers. Bottom panels show Western blots from the same set of samples using indicated antisera. (B) Top panel is Coomassie Blue stained gel loaded with equal cell number per lane. The amount loaded was not recalibrated after mitosis and represents equal mother cell equivalents. Bottom panels show Western blots from the same set of samples using indicated antisera. (C,D) Quantitation of Western signals from (A,B) respectively. CB staining was used to normalize in (C), while HSP70B signal was used to normalize in (D). Normalized data were plotted relative to the signal at 0 hrs that was set to a value of 1. (E) Fraction of flagellated cells as in Fig. 1B.

Using the same synchronous cultures as described above, whole cell protein samples were prepared at different time points throughout the cell cycle, and the levels of IFT proteins were examined by western blotting. The protein gels were loaded in two ways in order to highlight the cyclical nature of IFT protein expression. In Fig. 3A each lane was loaded with an equal amount of whole cell protein, whereas in Fig. 3B each lane was loaded with protein extract from an equal number of cells. The two loading methods differed due to the six to eight-fold size increase of each cell during G1. When loaded by equal protein per lane (Fig. 3A) the number of cell equivalents in each lane gradually decreased from 0–12 hours because each cell contributed more protein as it enlarged. Western blotting was used to determine the accumulation patterns of IFT proteins that were normalized to show relative concentration (Fig. 3A), or abundance on a per cell basis (Fig. 3B). Results were plotted in Fig. 3C and 3D respectively along with the fraction of flagellated cells (Fig. 3E).

When gel loading was normalized for equal protein per lane, IFT27 and IFT46 band intensities on western blots decreased during G1 (0–12 hours) while FLA10 levels remained relatively constant (Figs. 3A and 3C). This difference between the accumulation patterns of IFT27/46 and FLA10 proteins is consistent with their mRNA accumulation patterns, where the mRNAs corresponding to IFT27 and IFT46 show much lower expression in G1 than they do during S/M, while FLA10 mRNA is less stringently controlled (Fig. 2). During S/M, there was a sharp increase in IFT27 and IFT46 proteins that restored their amounts to just above their starting point at the beginning of G1 (Fig. 3A and 3C). The decrease in IFT27 and IFT46 protein concentrations during G1 shown in Fig. 3A was attributable to dilution rather than breakdown because IFT amounts per cell remained constant during G1 while the total amount of protein per cell increased. Further illustrating this point are results from gels loaded with equal cell numbers per lane (Fig. 3B and 3D). Here, IFT27 and IFT46 band intensities remained relatively constant throughout G1, while an HSP70B control band increased in intensity during G1 as total cytoplasmic protein per cell increased. Thus, as highlighted in Figs. 3A and 3C, the concentration of IFT proteins within each cell constantly decreased as cells enlarged during G1, and they were at their lowest concentration just prior to S/M. In contrast to the tight cell cycle regulation of IFT27 and IFT46 proteins, FLA10 concentration remained nearly constant with respect to total protein in the cell, suggesting that FLA10 synthesis was ongoing during most of G1 and did not display a strong cell cycle regulatory pattern. Key experiments described in Figs. 1,2,3 were repeated with a different wild-type strain, CC125, and yielded comparable results (Fig. S2).

**Figure 4:**
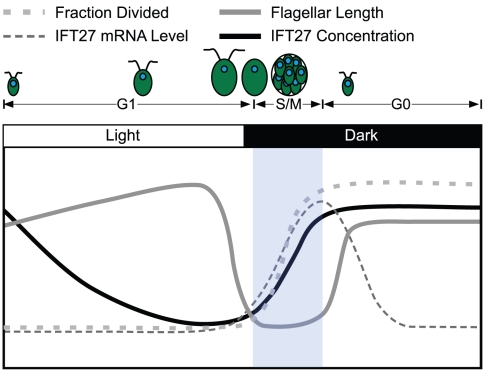
Summary of IFT27 protein and mRNA synthesis during the cell cycle. The relative concentrations of IFT27 protein (solid dark line) and IFT27 mRNA (thin dashed line) are plotted along with flagella length (solid gray line) and progression through S phase and mitosis (thick dashed line). IFT27 protein concentration drops continuously during G1 and is at its lowest just before division. IFT27 mRNA and protein are normally synthesized during S/M which resets its levels for the next cell cycle.

Taken together, our data indicate that each daughter cell begins with an allotment of IFT27 and IFT46 whose amount per cell remains constant during G1, and that the IFT proteins do not normally accumulate again until the next round of cell division (S/M) is completed. The simplest explanation for this pattern is that IFT proteins are only synthesized once per cell cycle as cells exit S/M and then remain relatively stable throughout G1, an interpretation that is also consistent with the mRNA abundance profiles. However, a more complicated possibility is balanced synthesis and turnover that maintains a constant steady state abundance of IFTs during G1. In either case, a consequence of this expression pattern is that the concentration of IFT per total cell protein decreases as cells enlarge several-fold in size during G1. The patterns of IFT mRNA and protein accumulation during the cell cycle are summarized in Fig. 4.

### Subcellular localization of IFT proteins during the cell cycle

**Figure 5:**
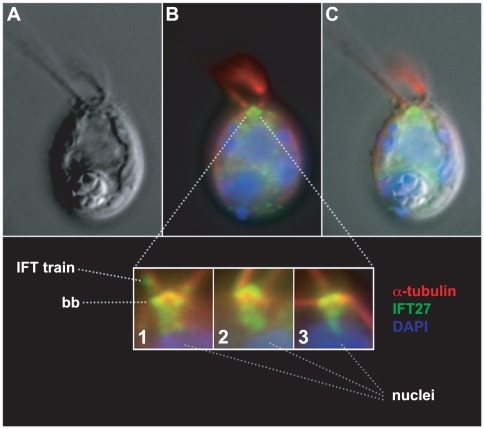
Location of IFT27 during interphase. (A) Representative interphase cell imaged with DIC microscropy. (B) The cell in panel A was subjected to widefield indirect immunofluorescence microscopy with antibodies specific for IFT27 (green) and α-tubulin (red). The nuclear and chloroplast DNA are stained with DAPI (blue). (C) Merged image from panels A and B. Inset panels 1–3 display overlays of immunofluorescence and DAPI signals at the basal body regions of three different cells at three different angles of rotation with respect to the longitudinal axis of the cell body. The location of basal bodies (bb), an IFT train, and the upper edges of DAPI stained nuclei are marked by dotted lines. A prominent structure extending between the basal bodies and nuclei is revealed by IFT27 immunofluorescence in each cell.

To gain further insight into the cyclical regulatory pattern of IFT proteins, their subcellular location during interphase was compared to their location at the time of cell division. Polyclonal antibodies raised against IFT27 were used for immunofluorescence localization with synchronous cell populations. Previous studies showed localization of most of the interphase IFT proteins at the basal bodies with a smaller amount comprising the IFT particle trains found between the axoneme and flagellar membrane [5], [11], [41], [42]. In the present study, we found IFT27 at the basal bodies, and in the flagella during interphase (Fig. 5). In addition, a prominent trail of IFT27 fluorescence was found extending between the basal bodies and the anterior surface of the nucleus. Fig. 5B inset panels 1–3 display representative examples of this pattern of fluorescence taken from three different cells at three different angles of rotation with respect to the longitudinal axis of the cell body.

**Figure 6:**
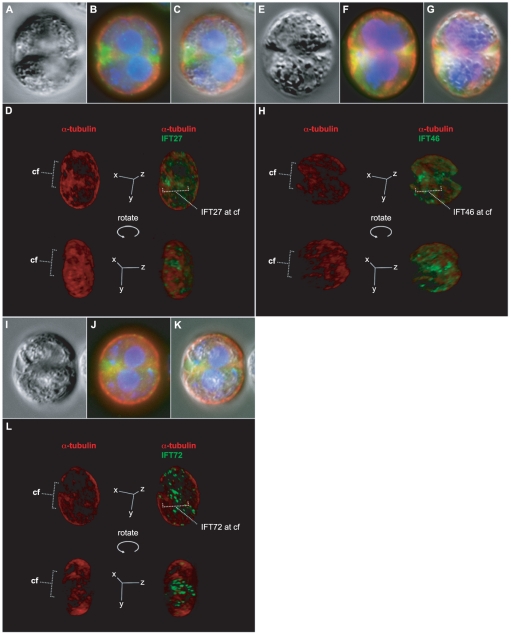
Location of three IFT27, IFT46 and IFT72 during cleavage furrow formation. (A,E,I) DIC micrographs of three different dividing Chlamydomonas cells at the time of first cleavage furrow formation. (B,F,J) The immunofluorescence locations of α-tubulin (red), DAPI stained DNA (blue), and the indicated IFT protein (green) observed by widefield microscopy. (B,C) IFT27. (F,G) IFT72. (J,K) IFT46. (D,H,L) 3D reconstructions of optical sections obtained by laser scanning confocal microscopy of three additional dividing Chlamydomonas cells that were subjected to indirect immunofluorescence labeling at the time of cleavage furrow formation. Appearing in red is a reconstruction of each cell's microtubule cytoskeleton shown in side-view at the upper left and right of each panel. The locations of the cleavage furrows (cf) are marked by dotted lines. The 3D reconstructions are rotated about the y-axis giving a view directly into the cleavage furrows in the images shown at the bottom of each panel. The immunofluorescence signals corresponding to IFT27, IFT46 and IFT72 are included and shown as green to the right in panels D, H and L respectively. All three IFT proteins are clustered at the cleavage furrow.

**Figure 7:**
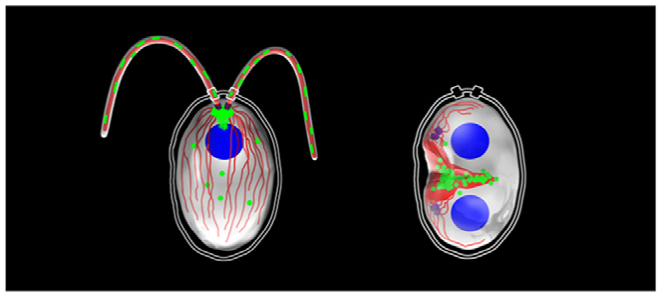
Summary of IFT localization. The subcellular location of IFT27 and other IFT complex B proteins are summarized in a cartoon diagram. The left image depicts an interphase cell where IFT27 (green) is found concentrated principally at the basal bodies and on IFT trains in the flagella (microtubules are drawn in red). In addition, IFT27 is found on a structure located between the basal bodies and the nucleus (blue) and on diffuse puncta in the cytoplasm during interphase. The right image depicts a dividing cell at the time of cleavage furrow formation. Here, IFT27 and other IFT complex B proteins are no longer associated with the basal bodies (purple), and instead cluster along the nascent furrow.

As Chlamydomonas cells transition from interphase to S/M, a series of morphological rearrangements take place [28], [29], [38], [48], [49], [50], [51]. Following the loss of flagella, the protoplast undergoes a 90-degree rotation with respect to its surrounding cell wall. The original anterior surface of the cell is marked by the location of two cell wall channels through which the flagella had passed during interphase [38], [50] (illustrated in Fig. 7). At the first mitotic division, the cleavage furrow begins by extending inward on one side of the cell in a plane transverse to that of the cell wall channels. Then the furrow extends circumferentially around the cell as it penetrates medially into the cell's interior. The subcellular location of IFT27 during this process is striking. Figure 6A shows a DIC micrograph of a dividing cell as it forms its first cleavage furrow. Figure 6B shows this same cell subjected to immunofluorescence labeling with antibodies specific for α-tubulin (red) and IFT27 (green), and stained with DAPI to reveal the position of daughter nuclei (blue). An intense concentration of IFT27-specific fluorescence is observed at the forming cleavage furrow, and the pattern of fluorescence observed at the basal bodies during interphase is gone.

Laser scanning confocal microscopy was used to generate a 3D reconstruction of the α-tubulin cytoskeleton in a cell at the time of cleavage furrow formation (Figure 6D). The structure of the furrow is delineated by a concentration of α-tubulin signal in red. The same cell is shown at the top right with the location of IFT27 displayed in green. Rotating the 3D reconstruction gives a view directly into the cleavage furrow where IFT27 appears to line its inner surface (Figure 6D, Movie S1). A similar pattern of localization at the cleavage furrow was documented for two additional IFT complex B proteins, IFT46 (Figure 6 E–H) and IFT72 (Figure 6, I–L); as well as an IFT complex A protein, IFT139 (Figure S4), suggesting that this pattern is characteristic of both IFT subcomplexes during cytokinesis. An antibody specific for the intermediate chain of Chlamydomonas outer arm dynein IC69, a structural component of the flagella, gives a prominent localization in flagella of interphase cells, but is not found associated with IFT proteins at the cleavage furrow during division (Figure S4).

As the furrow matures and daughter cells prepare to separate, IFT fluorescence begins to leave the furrow and regroup back at the basal bodies prior to growth of new flagella (Figure S3). Because the amounts of IFT27 mRNA and protein increase during division, it is possible that newly synthesized IFT27 and other IFT proteins may first localize around the cleavage furrow prior to arriving at their interphase destination at the basal bodies and flagella. The localization patterns of IFT complex B proteins is summarized in Fig. 7.

**Figure 8:**
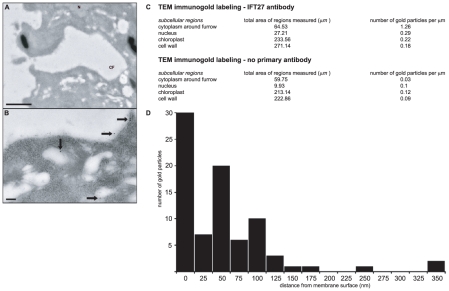
TEM immunogold labeling of IFT27 during cleavage furrow formation. (A). A representative TEM micrograph of a forming cleavage furrow (CF) is shown at a magnification of 12,000×. A nucleus is marked (N) near the top, and numerous membrane vesicles are observed surrounding the furrow. The dark stained structures are starch granules. A scale bar representing 1 µm is placed at the lower left. (B) A region from the same section as A is shown at higher magnification (80,000×) so that IFT27-specific, 12 nm gold particles are visible. Five particles appear in this image and are marked by black arrows (the arrow at upper right points to two gold particles). Each 12 nm particle is directly adjacent to a membrane surface. Smaller 6 nm gold particles seen here are specific for α-tubulin. A scale bar representing 100 nm is placed at the lower left. (C) The total number of gold particles found and photographed in seven individual cell sections, labelled with IFT27-specific antibodies, were counted and categorized by their subcellular location within the cytoplasmic region around the furrow, nuclei, chloroplasts, or cell walls. Shown in the upper table is the total area of each of these regions and the number of gold particles found there per square micron. The lower table shows the results of a control experiment in which the process was repeated for an additional seven cell sections that were subjected to labeling in the absence of primary antibody. (D) The distance between each of the gold particles found in the cytoplasmic region, as quantified in the upper table in C, and the nearest cytoplasmic vesicle or plasma membrane surface was measured and displayed here as a distribution.

To determine the precise subcellular location of IFT27 at the cleavage furrow, dividing Chlamydomonas cells were analyzed by immunogold labeling and transmission electron microscopy (TEM). Figure 8A shows a representative image of a forming cleavage furrow (CF). A nucleus is marked (N) and numerous vesicles are observed surrounding the furrow. Figure 8B shows a higher magnification of the innermost region of the furrow depicted in 8A. Five IFT27-specific, 12 nm gold particles are observed and marked with black arrows. Each gold particle is immediately adjacent to either a vesicle or furrow membrane surface. Most gold particles detected in our experiments were found on membrane vesicles in the cytoplasm surrounding cleavage furrows with a lower background level of labeling in nuclei, chloroplasts and cell walls. Figure 8C tabulates the number of gold particles found per square micron in each of these subcellular regions measured across seven sections through different dividing cells. The distance between each gold particle and the nearest membrane surface was measured for all gold particles found in the region of cytoplasm surrounding the cleavage furrow. The distribution of these measurements is displayed in Figure 8D. These findings show that the IFT27-specific immunofluorescence signal observed at the cleavage furrow is indicative of an association with vesicles.

## Discussion

Coordination between ciliogenesis and the cell cycle has been observed for decades, but very little is known about how it is achieved [1], [32], [39], [40]. Here we have documented the expression pattern of IFT proteins, required for ciliogenesis, during the cell cycle and we have identified a novel localization pattern for IFT complex B proteins at the cleavage furrow.

Rather than reach a steady-state length, flagella elongate throughout G1 as cells increase in size, and our measurements with synchronized Chlamydomonas cultures indicate a correlation between flagellar length and cell size that has not been previously described in detail, though our data are consistent with previous observations of gradual lengthening of interphase flagella in Chlamydomonas [52] and in the protist Peranema trichophorum [53]. The amounts of IFT27, IFT46 and presumably other IFT proteins, per cell, do not increase during interphase. Instead, their concentration per total cell protein decreases as the cell progresses through interphase and enlarges several fold (Figs. 3 and 4). Thus, the lengthening of flagella observed during G1 continues to occur while the total concentration of IFT per cell is decreasing.

### Control of IFT mRNAs during the cell cycle

Transcriptional control of cell cycle mRNAs is well documented [54], [55], [56], [57], though to our knowledge only one other study in Trypanosomes has examined the relationship between mRNAs for flagellar proteins and cell cycle phasing [58]. In Chlamydomonas the expression of mRNAs encoding core cell cycle regulatory proteins is repressed during G1 and induced to high levels during S/M [46]. We have shown here that IFT mRNAs follow a similar pattern to cell cycle mRNAs, but their accumulation is phase shifted by about two hours (Fig. 2C). The multiple fission cell cycle of Chlamydomonas can produce up to 32 daughters in a single cell cycle [59], and in our culture system 8 daughters per mother cell is routine (Fig. 1). The coupling of IFT mRNA and protein levels to the rapid burst of division cycles during S/M provides a means for cells to increase their IFT protein complement to accommodate the requirement for production of new flagella on each daughter. Previously, the only known trigger for IFT mRNA accumulation was found to be deflagellation [60]. Here we have shown the regulatory pattern of IFT mRNAs during the cell cycle, which involves a precisely timed accumulation that coincides with the completion of cytokinesis and flagella regrowth. It will be interesting to determine how the cell cycle-dependent accumulation of IFT mRNAs relates to their stress-induced accumulation as a consequence of deflagellation, and whether these two processes share common regulatory inputs.

### IFT protein levels during the cell cycle

IFT complex B protein levels followed a similar pattern as their corresponding mRNAs, with a marked absence of protein accumulation until S/M. Thus, each daughter begins with a complement of IFT proteins that does not change in amount per cell during the subsequent long G1 period even as the cells grow several-fold in size, thereby causing the IFT concentration to decrease as the amount of total protein increases. (Figs. 3 and 4). Nevertheless, throughout G1, flagellar length slowly increases. IFT complexes in the cell body cluster around basal bodies, so their local concentration may be sufficient to permit slow flagellar length increase during G1 as the concentration of IFT per total cell protein is decreasing. Our data indicate that the abundance of IFT proteins is tightly controlled and that these proteins are not normally synthesized until cell division. A second type of regulation may occur at the level of IFT subcomplex assembly. In Chlamydomonas, IFT27 is a global regulator of both IFT complex A and IFT complex B subunit abundance. When IFT27 amounts are reduced by RNAi, concomitant reductions are observed in IFT proteins of both complex A and B [24]. IFT27 might, therefore, be used to set the levels of the other IFT proteins.

### Localization of IFT at the cleavage furrow

We have documented a novel localization pattern for IFT proteins IFT27, IFT46, IFT72, and IFT139 in which they are repositioned from their interphase location at basal bodies and flagella to the cleavage furrow at the time of cell division. During interphase, IFT27 immunofluorescence shows a localization pattern at the basal bodies similar to that reported previously for IFT46, another IFT complex B protein [42]. The affinity purified IFT27 antibody used in this study did not produce a large, intensely staining area distal to the basal bodies as reported previously [43]. We believe that this localization by Wang et al. was due to an antibody impurity, because the signal disappeared from our immunofluorescent staining when these same antibodies were stringently purified. Also not documented previously is the prominent pattern of IFT27-specific fluorescence between the basal bodies and the nucleus shown here in Figure 5B. (inset panels 1–3).

At the time of cleavage furrow formation in Chlamydomonas, IFT27, IFT46, IFT72, and IFT139 are no longer localized prominently to the basal body region by immunofluorescence, and instead are found clustered around the furrow region (Figs. 6, 7; Fig. S4). The localization pattern of these proteins is seen returning to the basal bodies in cells observed at later stages, in which the furrow has matured and cells are completing cleavage (Fig. S3, and data not shown). Another flagellar protein, the intermediate chain of outer arm dynein (IC69), does not localize around the cleavage furrow at the time of division, indicating that the localization pattern observed for IFT is not a general property of all flagellar proteins (Fig. S4). Therefore, during interphase, IFT proteins form an accumulation at the basal bodies, from which IFT trains are derived, and function in the maintenance of flagella. At the time of cell division, when the flagella are resorbed, IFT complex B proteins are redistributed from the basal bodies to the forming cleavage furrow. Once the furrow has matured, the IFT proteins reform in a pool at the basal bodies prior to growth of new flagella.

Using immunogold labeling and TEM, the location of IFT27, at the time of cell division, was pinpointed predominantly to the surfaces of membrane vesicles surrounding the cleavage furrow (Fig. 8). The addition of membrane to the growing cleavage furrow is a process that could involve the IFT proteins whose furrow localization we report here. Multiple independent studies have begun to coalesce into a new picture of IFT as a regulator of membrane dynamics. The localization of IFT20 at the Golgi and its function in the sorting and trafficking of membrane proteins to the cilium is well-documented [61], [62], [63]. Proteins making up the core of IFT particle complexes have structure and sequence similarities to membrane vesicle coat proteins, such as COPI and clathrin [64], [65], [66]. The homology between IFT particles and vesicle coats suggests that IFT may have evolved from an intracellular coated vesicle transport process. Recent studies with T-lymphocytes have substantiated this notion by providing evidence that in a non-ciliated cell, IFT proteins can form an intracellular complex that regulates membrane trafficking and is essential for signaling at the immune synapse [44], [67]. Further parallels between the immune synapse, cilia and cytokinetic furrow were described in a recent review [68]. Another example of a function for IFT proteins in membrane trafficking in non-ciliated cells was described by a combined high resolution immunofluorescence and immunoelectron microscopy approach that revealed a novel IFT localization in nonciliated retinal neurons in which subsets of IFT proteins are found associated with what appear to be cytoplasmic membrane vesicles at the postsynaptic terminal region [45]. The cleavage furrow localization of IFT we document here is therefore congruous with an emerging awareness of the role of IFT proteins in intracellular membrane trafficking in addition to their well-established roles in ciliary assembly.

## Materials and Methods

### Strains and cell culture synchronous conditions

Wild-type Chlamydomonas reinhardtii strains 21 gr or CC125 were used for experiments (Chlamydomonas Genetic Center, Duke University, Durham, NC). Cells were grown phototrophically in high-salt medium (HSM) [69] and synchronized as follows: 300 mL cultures in 500 mL flasks were grown at 24C and bubbled continuously with 0.5% CO_2_ in air under a 12 hr∶12 hr light∶dark regime with 250 µm m^−2^ s^−1^ white light illumination provided by a combination of fluorescent bulbs (2 each, GE F40-SP65 and F40-C75). Synchrony was judged by mitotic index (fraction of dividing cells). Cultures where close to 100% of cells initiated their first mitosis within a two-hour window were used. Culture density was kept between 10^5^ and 10^6^ cells ml^−1^.

### Cell cycle progression and flagellar length

Samples were collected at intervals from cultures at densities of 3–5×10^5^ ml^−1^ during a single 24 hour period (one full cycle) and prepared as follows: For cell size, mitotic index and flagella length, 1 mL of cells was fixed in 0.2% glutaraldehyde, 0.005% Tween 20 (final concentrations). Cell size and density were determined using a Coulter Counter with a 100 µm diameter aperture (Multisizer 3; Beckman-Coulter, Miami, Florida, United States). Division number was determined by microscopic examination of individual cells spread onto thin HSM agar plates and left in the dark. Cells or clusters of post-mitotic cells were examined using a Zeiss Axiostar Plus microscope with a 40× objective and phase contrast optics. Single cells and those that had divided once, twice and three times were determined by examining at least 300 cells per time point. The period of division (S/M) was determined as the time window between the appearance of the first divided cells (∼13 hours) and the end of any additional divisions (∼17 hours). Flagella lengths of unhatched daughters were determined by pre-treating post-mitotic mother cells with autolysin to release the daughters from the mother cell wall (Harris, 1989). Released daughters were fixed as described above. Flagella images were taken with a light microscope (Nikon Eclipse TE2000 microscope equipped with a 100×, 1.4NA objective lens), and flagella lengths were measured using a manual curve tracing function in Metamorph v6.1 (Molecular Devices). For each time point, about 200 flagella were measured. Cell sizes for individual cells in Figure S1 were calculated as described previously [70]. Resampling to generate 100,000 randomly permuted replicates of the data and linear regression were done using the open source program R [71] and the package Boot [72].

### Protein sample preparation and antibodies

Rabbit antibodies for IFT27, IFT46, FLA10 and HSP70B were used as described previously [24]. The IFT27 antibody used here was affinity purified rabbit antibody against full length IFT27 tagged with maltose-binding protein (MBP).

For Figure 3A, 15 mls of cells were pelleted and resuspended in freshly prepared 0.1 M Na2CO3, 0.1 M DTT. They were immediately lysed by adding 0.66 volumes of 5% SDS, 30% sucrose. Debris was pelleted by centrifugation at 16,000 g (Eppendorf 5415C) and protein content of supernatants was determined using the amido black assay calibrated with BSA as described previously (Huang et al., 2002). 10 µg of total protein in 1X SDS-PAGE sample buffer was loaded in each lane. During G1 (hours 0–12) protein per cell increased by about eight fold, so fewer cells were loaded per lane to give a consistent amount of protein per lane at each successive time point up until cell division.

For Figure 3B protein samples were prepared by spinning down 1.5 mls of cells at each time point followed by lysis in 50 µl 1X SDS-PAGE sample buffer. Cell number remained constant for interphase cells and then increased by about eight-fold during mitosis. 1.5 ml samples continued to be collected for post-mitotic cells in order to maintain equivalent loading. 15 µl of lysate was loaded per lane.

Samples were electrophoresed through 10% SDS-PAGE gels. Western blots were carried out as described previously [43]. Western band intensities were determined by digital scanning of film and quantitation in Adobe Photoshop using background corrected mean intensities. After normalization to CB staining in Fig. 3A or to the HSP70B signal in Fig. 3B the signals were calibrated to the 0 hr time point that was set at an intensity of 1.

### RNA preparation and quantitative RT-PCR

Approximately 1–2.5×10^7^ cells were pelleted at each time point. RNA and cDNA were prepared as described previously [47]. Quantitative RT-PCR was performed as described previously (Fang et al., 2006) using the primer pairs listed in Table S1 and the following program: 94 C 3 min, 40 cycles of 94 C 10s followed by 63 C for 40s. PCR reactions were performed in triplicate samples from each of two independent biological replicates. Quantitation was normalized to the internal control gene GBLP and based on the method described in [73].

### Immunofluorescence microscopy

Wild type Chlamydomonas were fixed for 1 hour at room temperature in MI growth medium containing 1% paraformaldehyde. The cells were then washed with phosphate buffered saline (PBS) and pipetted onto 0.1% polyethyleneimine-coated cover slips where they sank to the surface and attached over the course of 20 minutes at room temperature. To permeabilize and fix additionally, cover slips with attached cells were submerged in methanol at −20 C for 5 minutes, then transferred to fresh methanol for an additional 5 minutes at −20 C. Cells were rehydrated for 2 minutes in PBS then incubated at room temperature for 1 hour in blocking buffer (5% BSA, 1% cold water fish gelatin, and 10% goat serum in PBS). Cells were overlayed with a mixture of primary antibodies in blocking buffer and incubated in a moist chamber at 4 C overnight. After washing away primary antibodies by repeatedly dipping the coverslips in PBS, cells were overlayed with a mixture fluorophore-conjugated secondary antibodies (Alexa Fluor 488 goat anti-rabbit IgG and Alexa Fluor 594 goat anti-mouse IgG, Invitrogen Molecular Probes) in blocking buffer and incubated at room temperature for 1 hour. Coverslips were washed by dipping in PBS as before and mounted to slides with SlowFade Antifade reagent (Invitrogen Molecular Probes). Laser scanning confocal microscopy was performed using a Zeiss LSM-510 system with an Axio Observer inverted microscope. 3D reconstructions were performed using Zeiss Zen software and individual images were adjusted using Adobe Photoshop. Widefield fluorescence microscopy was performed using a Nikon Eclipse TE2000-U inverted microscope with a Photometrics CoolSNAP HQ CCD camera. Images were collected using Molecular Devices MetaMorph software and adjusted using Adobe Photoshop.

### Postembedding immunogold labeling and transmission electron microscopy

Chlamydomonas reinhardtii, cc125, were harvested from synchronously dividing cultures by centrifugation at 500 g. Cell pellets were resuspended in M1 medium (Harris, 1989) and fixed by addition of 4% formaldehyde an 0.5% glutaraldehyde for 1 hour at room temperature. Following two washes in MT buffer (30 mM HEPES, 5 mM Na-EGTA, 15 mM KCl, pH 7.0), cells were incubated in 0.04% osmium tetroxide on ice for 30 minutes. Cells were pelleted at 500 g and pellets were washed three times with distilled water then embedded in 1% agar prior to dehydration to 100% ethanol (15 minute incubations each of 30% and 50% ethanol on ice; then 70%, 95%, and 100% ethanol at 20°C). Infiltration of the sample with LR Gold resin (Plano, Marburg, Germany) was performed at −20°C according to the following scheme: 0.4% Benzil acitivated LR Gold/ethanol (1∶1) for 3 hours followed by transfer of the sample to 0.4% Benzil acitivated LR Gold alone for 24 h. Polymerization was performed under fluorescent light for 24 h at −20°C. Ultrathin sections (60 to 80 nm) were cut with a diamond knife (Diatome, Biel, Switzerland) on an Reichert Ultracut E Ultramicrotome (Reichert Microscope Services, Depuy, New York) and collected on formvar coated nickel grids. The sections were subjected to a saturated solution of sodium metaperiodate for 4 minutes, washed four times for 5 minutes each with distilled water, then washed two times for 5 minutes each in phosphate-buffered saline [PBS]. Blocking was performed for 1 hour at room temperature with blocking buffer (5% BSA, 1% cold water fish gelatin, and 10% goat serum in 1× PBS in phosphate-buffered saline [PBS]; pH 7.4). Incubation of grids in primary antibody mixture, anti-IFT27 and anti-α-tubulin in blocking buffer was performed overnight at 4°C alongside control experiments in which block buffer alone was added with no primary antibodies. Grids were washed five times with PBS for 10 min each and incubated for 1.5 hours in secondary antibody mixture, 6 nm gold particles conjugated to goat anti-mouse IgGs, and 12 nm gold particles conjugated to goat anti-rabbit IgGs (Jackson ImmunoResearch, West Grove, PA). Grids were washed five times with PBS for 10 min each, fixed for 7 min in 1% glutaraldehyde in PBS, and washed three times for 10 min each with distilled water. Specimens were stained with lead citrate for 2 minutes and photographed with a JEOL JEM-1230 electron microscope (JEOL USA, Inc. Peabody, MA).

## Supporting Information

Figure S1 **Correlation between cell size and flagella length.** (A) log-log scatter plot of ∼200 flagella length and cell size measurements from individual cells (blue points) taken from two independent asynchronous cultures (∼100 cells each). The best fit linear regression is plotted as a dashed gray line. The regression line function and its R^2^ value are shown above. (B) Histogram plot of R values from 100,000 randomized re-sampled sets of data from A. Mean R value and standard deviation (SD) for resampled data are shown. The actual R value is indicated by an arrow. The p-value of obtaining this result from the randomly resampled distribution is shown below it.
(EPS)

Figure S2 **Biological replicate of IFT protein accumulation during synchronous division.** (A) Cultures of wild type strain CC125 were synchronized to a 12∶12 light dark cycle and cell divisions were plotted as in Fig. 1. S/M started earlier in CC 125 than in 21 gr (Fig. 1), but the relative timing of mRNA and protein expression for IFTs was similar. (B) Quantitative RT-PCR of samples from synchronized CC125 plotted as described in Fig. 2. (C) Coomassie Blue (CB) stained protein gel (top panel) or Western blots (lower three panels) from SDS-PAGE gels loaded with samples of synchronized CC125 with equal protein per lane similar to Fig. 3C. Antibodies are indicated to the left of each panel and cell cycle time points are indicated below each lane. The time points encompassing S/M are shaded light blue.
(EPS)

Figure S3 **IFT27 re-accumulates at the basal bodies after cleavage furrow formation.** Two examples are shown of Chlamydomonas, cell 1 (A–E) and cell 2 (F–J), subjected to widefield immunofluorescence localization during the final stages of cell division following cleavage furrow formation. An accumulation of IFT27-specific fluorescence can be seen returning to the basal body regions, marked by arrows, in cell 1 (A, E) and in cell 2 (F, J), while residual amounts of IFT27 can still be observed lining the central area where the cleavage furrows had formed. DIC micrographs of each cell are shown (D, I), and α-tubulin specific fluorescence (B, G) and DAPI-stained DNA images (C, H) are superimposed over the DIC micrographs (E, J).
(EPS)

Figure S4 **IFT139 localizes to the cleavage furrow but the structural flagellar dynein IC69 does not.** (A) Four examples of dividing cells are shown with their DIC images depicted at the left and each cell's corresponding fluorescence localization image shown at the right. The cleavage furrow of cell 1 (panel A1) is indicated by a dotted mark; all other cells are oriented similarly. Nuclei are stained with DAPI and displayed in blue. IFT139 localization appears in green. IFT139 appears concentrated in the cleavage furrow of each cell. (B) Four dividing cell examples are arranged as those in (A). IC69 localization appears in green. IC69 is absent from the cleavage furrow. (C) Two flagellated, interphase cells were treated as in (B). IC69 is observed throughout the flagella in green. Nuclei and chloroplast DNA are stained with DAPI in blue.
(EPS)

Movie S1 **Rotating view of IFT27 and microtubule cytoskeleton during cleavage.** The cell is the same as that shown in Fig. 6D.
(MOV)

Table S1 **Primers used for RT-PCR.**
(DOCX)

Table S2 **Data for quantitative RT-PCR.**
(DOCX)

Text S1 **Relationship between cell size and flagellar length.**
(DOCX)
