# Supplementary material for: IFT Proteins Accumulate during Cell Division and Localize to the Cleavage Furrow in Chlamydomonas
Source: PLoS One. 2012 Feb 6;7(2):e30729. doi: 10.1371/journal.pone.0030729 (PMC3273483; doi:10.1371/journal.pone.0030729)
Supplement: Table S1 — Primers used for RT-PCR. (DOCX) [file pone.0030729.s006.docx]

Table S1. Primers used for RT-PCR

| **Gene Name** | **JGI ID^a^** | **Primer** | **Sequence 5’->3’** |
| --- | --- | --- | --- |
| *IFT27 (FAP156)* | 129193 | 27QPF2 | TCTCGGTGGAGCTCTTTCTG |
|  |  | 27QPR3 | GCTCACATCGAACACGAGAA |
| *IFT46* | 520197 | 46QPF1 | TGACATAGGAGCAAGCAGCA |
|  |  | 46QPR1 | CTCCGACTCACTCAGGTTCA |
| *IFT140* | 192205 | 140E26F | CAACTACCTGCAGACGCTCA |
|  |  | 140E27R | CGGTACTCGTCCACCTCAAT |
| *FLA10* | 185750 | Fla10F1 | CCATCGAGAAGGAGAAGCAG |
|  |  | Fla10R1 | GTCCACCTTTTCCAGCATGT |
| *GBLP* | 164254 | GBLP3 | GTCATCCACTGCCTGTGCTTCT |
|  |  | GBLP4 | GGCCTTCTTGCTGGTGATGTT |
| *CYCB1* | 206115 | CYCB1-3 | GGTGCACCTGAAGTTCAAGCTG |
|  |  | CYCB1-4 | AATCTCCTCGTACTTGGACGCG |

**a** Protein ID from JGI Genome Browser: http://genome.jgi-psf.org/Chlre4/Chlre4.home.html
