# Supplementary material for: IFT Proteins Accumulate during Cell Division and Localize to the Cleavage Furrow in Chlamydomonas
Source: PLoS One. 2012 Feb 6;7(2):e30729. doi: 10.1371/journal.pone.0030729 (PMC3273483; doi:10.1371/journal.pone.0030729)
Supplement: Table S2 — Data for quantitative RT-PCR. (DOCX) [file pone.0030729.s007.docx]

Table S2. Data for quantitative RT-PCR.

|  | ***IFT27*** | | ***IFT46*** | | ***CYCB*** | | ***IFT140*** | | ***FLA10*** | | ***GBLP*** | |
| --- | --- | --- | --- | --- | --- | --- | --- | --- | --- | --- | --- | --- |
| **Time (hrs)** | **CT** | **SD** | **CT** | **SD** | **CT** | **SD** | **CT** | **SD** | **CT** | **SD** | **CT** | **SD** |
| 0 | 26.067 | 0.349 | 27.060 | 0.372 | 32.130 | 0.131 | 28.503 | 0.221 | 28.203 | 0.207 | 17.510 | 0.404 |
| 2 | 34.330 | 0.667 | 32.455 | 0.403 | 33.057 | 0.424 | 34.007 | 0.219 | 29.733 | 0.187 | 17.730 | 0.576 |
| 4 | 26.003 | 0.067 | 27.613 | 0.042 | 33.353 | 0.348 | 28.830 | 0.140 | 26.847 | 0.129 | 18.050 | 0.533 |
| 6 | 26.247 | 0.546 | 27.163 | 0.671 | 30.550 | 0.279 | 28.877 | 0.040 | 26.120 | 0.165 | 18.307 | 0.223 |
| 8 | 25.510 | 0.340 | 25.957 | 0.179 | 31.360 | 0.417 | 27.333 | 0.353 | 25.400 | 0.285 | 18.727 | 0.304 |
| 10 | 26.757 | 0.168 | 26.340 | 0.100 | 25.993 | 0.035 | 26.950 | 0.191 | 26.030 | 0.166 | 19.268 | 0.447 |
| 11 | 25.333 | 0.206 | 25.800 | 0.095 | 22.957 | 0.309 | 26.880 | 0.653 | 25.913 | 0.379 | 19.295 | 0.440 |
| 11.5 | 26.057 | 0.095 | 26.933 | 0.101 | 21.570 | 0.181 | 27.453 | 0.339 | 26.143 | 0.340 | 19.217 | 0.501 |
| 12 | 26.140 | 0.465 | 26.533 | 0.320 | 21.060 | 0.035 | 27.050 | 0.144 | 27.163 | 0.247 | 18.713 | 0.197 |
| 12.5 | 26.050 | 0.278 | 26.660 | 0.322 | 20.510 | 0.312 | 26.283 | 0.274 | 26.470 | 0.515 | 18.977 | 0.310 |
| 13 | 23.907 | 0.059 | 26.670 | 0.061 | 20.147 | 0.006 | 26.517 | 0.227 | 28.210 | 0.095 | 19.083 | 0.263 |
| 13.5 | 22.727 | 0.129 | 24.377 | 0.025 | 18.313 | 0.081 | 23.937 | 0.316 | 25.363 | 0.119 | 18.293 | 0.226 |
| 14 | 22.863 | 0.365 | 24.933 | 0.369 | 19.227 | 0.095 | 24.160 | 0.372 | 26.080 | 0.494 | 18.922 | 0.494 |
| 14.5 | 21.653 | 0.136 | 23.803 | 0.055 | 19.260 | 0.157 | 23.737 | 0.262 | 25.143 | 0.300 | 18.748 | 0.413 |
| 15 | 21.433 | 0.055 | 23.357 | 0.081 | 18.873 | 0.558 | 22.917 | 0.163 | 24.100 | 0.053 | 18.582 | 0.373 |
| 16 | 20.393 | 0.424 | 23.137 | 0.438 | 21.540 | 0.090 | 24.587 | 0.441 | 24.877 | 0.307 | 19.013 | 0.390 |
| 18 | 21.637 | 0.503 | 23.797 | 0.099 | 30.360 | 0.748 | 26.037 | 0.294 | 25.683 | 0.368 | 18.395 | 0.433 |
| 25 | 29.680 | 0.354 | 30.823 | 0.049 | 34.797 | 0.282 | 31.633 | 0.274 | 30.160 | 0.217 | 18.343 | 0.620 |

Average cycle time (CT) and standard deviation (SD) for each primer pair are shown for each amplicon
