# Supplementary material for: IFT Proteins Accumulate during Cell Division and Localize to the Cleavage Furrow in Chlamydomonas
Source: PLoS One. 2012 Feb 6;7(2):e30729. doi: 10.1371/journal.pone.0030729 (PMC3273483; doi:10.1371/journal.pone.0030729)
Supplement: Text S1 — Relationship between cell size and flagellar length. (DOCX) [file pone.0030729.s008.docx]

**Supporting Text S1**. The relationship between cell size and flagella length

To further understand the scaling relationship between cell size and flagella length data were collected from individual cells grown asynchronously in continuous light. When plotted on a logarithmic scale the cell size versus flagella length data fit a linear regression with a slope of ~.13 and an R^2^ of .2447 (Fig. S1A). The positive correlation between cell size and flagella length was also tested for 100,000 randomly resampled data sets (Fig. S1B). The correlation coefficients (R-values) from these resampled data clustered around a value of 0 with a p-value of <0.001 at R=.495 (.495 is the correlation coefficient for the real data). The relationship between cell size (V) and flagella length (F) follows a power law whose basis may be related to a physical parameter that correlates with cell size. However, the shallow slope coefficient of .13 for logV versus logF rules out proportional relationships based on cell volume (expected slope = 1.0), surface area (expected slope=0.67) or diameter/radius (expected slope=0.33).
